# Comparison of Cellular and Transcriptional Responses to 1,25-Dihydroxyvitamin D3 and Glucocorticoids in Peripheral Blood Mononuclear Cells

**DOI:** 10.1371/journal.pone.0076643

**Published:** 2013-10-08

**Authors:** Sonia S. Kupfer, Joseph C. Maranville, Shaneen S. Baxter, Yong Huang, Anna Di Rienzo

**Affiliations:** 1 Department of Medicine, University of Chicago, Chicago, Illinois, United States of America; 2 Department of Human Genetics, University of Chicago, Chicago, Illinois, United States of America; Harbin Institute of Technology, China

## Abstract

Glucocorticoids (GC) and 1,25-dihydroxyvitamin D3 (1,25(OH)_2_ D3) are steroid hormones with anti-inflammatory properties with enhanced effects when combined. We previously showed that transcriptional response to GCs was correlated with inter-individual and inter-ethnic cellular response. Here, we profiled cellular and transcriptional responses to 1,25(OH)_2_ D3 from the same donors. We studied cellular response to combined treatment with GCs and 1,25(OH)_2_ D3 in a subset of individuals least responsive to GCs. We found that combination treatment had significantly greater inhibition of proliferation than with either steroid hormone alone. Overlapping differentially expressed (DE) genes between the two hormones were enriched for adaptive and innate immune processes. Non-overlapping differentially expressed genes with 1,25(OH)_2_ D3 treatment were enriched for pathways involving the electron transport chain, while with GC treatment, non-overlapping genes were enriched for RNA-related processes. These results suggest that 1,25(OH)_2_ D3 enhances GC anti-inflammatory properties through a number of shared and non-shared transcriptionally-mediated pathways.

## Introduction

Glucocorticoids (GC) have been used as therapeutic agents in the treatment of a variety of immune-related diseases such as asthma, inflammatory bowel disease and psoriasis. GCs are steroid hormones that exert their primary effects through direct transcriptional mechanisms [1]. However, there is significant inter-individual and inter-ethnic variability in response to GC treatment [2]. Approximately 30% of individuals, especially those of African descent, have decreased response to GCs irrespective of disease type and severity [3–5]. *In vitro* lymphocyte GC sensitivity, measured by inhibition of mitogen-induced lymphocyte proliferation (% inhibition) of peripheral blood mononuclear cells (PBMCs), has been shown [6–13] to be a useful clinical predictor to steroids. We previously studied *in vitro* inhibition of lymphocyte proliferation in healthy donors and found a significant response to GC treatment overall (mean log_2_ fold change =-3.9, p=4.8 X 10^-15^) and a significant difference in inhibition of cellular proliferation between individuals of European and African descent (98.1% vs. 94.9%, p=0.018). Consistent with the notion that GCs act primarily through transcriptional mechanisms, we detected a significant correlation between transcriptional response and cellular inhibition within and between populations suggesting that differences in GC response partly reflect variation in transcriptional response [14].

While the role of GCs in immunity has been known for many years, the role of another steroid hormone, 1,25(OH)_2_ D3, in immune function has only recently gained attention [15]. *In vitro* studies show that both innate and adaptive immune responses in humans are targets of 1,25(OH)_2_ D3 [16–20]. Regarding inter-ethnic differences, it has been well established that, on average, African Americans have lower circulating serum levels of 25-hydroxyvitamin D (25(OH) D) compared to other US populations [21]. Moreover, African Americans have higher incidence and prevalence of diseases such as asthma, tuberculosis as well as colon and prostate cancers in which vitamin D is thought to play a protective role [22–24]. These observations have led to the hypothesis that differences in circulating vitamin D levels could contribute to different disease susceptibility. However, no previous study has investigated cellular and transcriptional responses to 1,25(OH)_2_ D3 treatment across populations.

Given variable responses to GCs and the immuno-modulatory effects of 1,25(OH)_2_ D3, there is interest in combining 1,25(OH)_2_ D3 and GCs in order to leverage this combination for more effective treatment especially in GC non-responders. Only a handful of studies have investigated the effects of combined GC and 1,25(OH)_2_ D3 treatment on the immune system. In plaque psoriasis, for example, combination treatment of 1,25(OH)_2_ D3 and topical steroids is more effective than either treatment alone [25]. In asthma, there is indirect evidence of synergistic effects in that pediatric asthmatics with higher serum 25(OH) vitamin D levels have better response to inhaled corticosteroid treatment [26]. These findings are supported by *in vitro* studies showing enhanced secretion of the anti-inflammatory cytokine IL-10 from regulatory T cells of steroid-resistant asthmatics treated with 1,25(OH)_2_ D3 [27]. In airway smooth muscle cells, 1,25(OH)_2_ D3 and GCs additively inhibited chemokine secretion lending further *in vitro* support to the idea that vitamin D treatment may enhance steroid therapy. Finally, a proteomics study focused on combination treatment in dendritic cell (DC) maturation and found that 1,25(OH)_2_ D3 dominated in inducing a tolerogenic DC profile [28].

While these data suggests vitamin D could serve as a useful adjunct in the treatment of immune-related disorders [29], the molecular processes that underlie this observation remain unclear. The additional effects of vitamin D treatment in individuals with weak responses to GCs could reflect differences in these transcriptional mechanisms, e.g. vitamin D effects on additional immune-related genes that are not regulated by GCs. In addition to providing an explanation for the adjuvant benefits of vitamin D, comparing the transcriptome-wide effects of these hormones could shed light on how each uniquely influences the immune system. Despite these benefits, there have been no systematic studies comparing transcriptome-wide responses to GCs and 1,25(OH)_2_ D3 in the same set of subjects. In order to learn about the molecular processes that enable vitamin D to enhance the immune-suppressive effects of steroids, we compared *in vitro* cellular and transcriptional responses to GC and 1,25(OH)_2_ D3 treatment. Importantly, we included both African-American (AA) and European-American (EA) subjects to determine if these effects differ across populations.

## Materials and Methods

### Ethics Statement

All donors to Research Blood Components (http://researchbloodcomponents.com) sign an IRB-approved consent form giving permission to collect blood and use or sell it for research purposes. Because the blood samples were not shipped to the University of Chicago with individually identifiable information, this study was not considered research using human subjects and did not require IRB review at the University of Chicago [per 45 CFR 46.102(d)].

### Subjects

We analyzed the same subjects (n=18) that were used for our previous study of cellular and transcriptional responses to dexamethasone [14]. All subjects were healthy donors to Research Blood Components and were not on any medications. Most samples were collected in the morning (0800 hours to 1200 hours, see Table S1). We recorded self-reported ethnicity, age, gender and date and time of blood drawing. There were 9 self-identified African Americans (AA) and 9 self-identified European Americans (EA) in this group. There were 10 males and 8 females, and the median age was 24.5 years (range 19.6-52.0 years). All samples for both the dexamethasone study and the current study were processed at the same time. Briefly, peripheral blood (100ml) from these subjects was obtained from Research Blood Components, and, within one day of collection, whole blood was shipped overnight at 4°C to the University of Chicago Human Immunological Monitoring Facility. Samples were processed in multiple consecutive batches. Serum 25-OH vitamin D levels were determined at the Clinical Chemistry Laboratory of the University of Chicago using a standard assay (cat no. 11875116160, Roche Diagnostics Corporation, Indianapolis, IN, USA). We previously measured cell composition in each blood sample using flow cytometry [14], which was expressed as % of total PBMCs for T cells (both cytotoxic and helper), B cells and monocytes. Proportions of each cell type are listed for each donor in Table S1.

### Cell culture and treatment

PBMCs were isolated from heparin-treated whole blood using a standard Ficoll protocol. PBMCs were washed in PBS and transferred to RPMI supplemented with 10% charcoal-stripped fetal bovine serum. Each sample was then divided into one aliquot of 1.8 x 10⁶ cells for proliferation assays and 9 x 10⁶ cells for genome-wide transcriptional profiling. PBMCs were seeded at 1 X 10^6^ cells/ml for all experiments.

### Proliferation assays

PBMCs were grown in 96-well plates with 2 X 10^5^ cells per well. We performed three replicates of the following treatment groups: 1) 100nM 1,25(OH)_2_ D3 + 2.5 µg/ml PHA, 2) EtOH (vehicle control) + 2.5 µg/ml PHA, and 3) no treatment. Cells were cultured for 48 hours and lymphocyte proliferation was measured by H^3^-thymidine incorporation as previously described [14]. Percent inhibition (%I) was calculated as 1-[(proliferation in vitamin D +PHA)/(proliferation in EtOH + PHA)]. Linear regression was used to test for the effects of covariates on %I and population differences were measured using a one-tailed t-test. For the 1,25(OH)_2_ D3 and dexamethasone single treatment studies, we performed proliferation assays for all 18 individuals. For the combined treatment of 1,25(OH)_2_ D3 and dexamethasone, we selected the 8 individuals total (6 AA and 2 EA) who were the least responsive to dexamethasone treatment and for whom sufficient PBMCs were available. Proliferation assays were all performed in the Human Immunological Monitoring Facility at the University of Chicago.

### Transcriptional response

We performed transcriptional profiling on 12 subjects (6 AA and 6 EA). PBMCs were grown in 24-well plates with 10⁶ cells per well. The following treatments were performed with three replicates per donor: 1,25(OH)_2_ D3 + PHA and EtOH + PHA. We included two time points: 8 hours and 24 hours. Replicates were then pooled and total RNA was extracted from the pool using the QIAgen RNeasy Plus mini kit. RNA was extracted from all 48 samples on the same day. Total RNA was reverse transcribed into cDNA, labeled, hybridized to Illumina (San Diego, CA, USA) HumanHT-12 v3 Expression BeadChips and scanned at the Southern California Genotyping Consortium at the University of California at Los Angeles. The microarray data has been deposited in the Gene Expression Omnibus (GEO), www.ncbi.nlm.nih.gov/geo, under accession number GSE50012. In order to minimize batch effects, all microarrays were hybridized on the same day and at the same time as the dexamethasone-treated samples. Summary data were obtained using the BeadStudio software from Illumina at the SCGC. Low-level microarray analyses were performed using the Bioconductor software package LUMI [30] in R. Probes were annotated by mapping to the RNA sequences from RefSeq using BLAT. Probes that mapped to multiple genes were discarded to avoid ambiguity in the source of a signal due to cross-hybridization of similar RNA species. Probes containing one or more HapMap SNPs were also discarded to avoid spurious associations between expression measurements and ethnicity due to inter-ethnic differences in allele frequencies. Variance stabilizing transformations were applied to all arrays, probes indistinguishable from background fluorescence levels were discarded and quantile normalization was done across all arrays. After these filters, 9,977 probes were used in subsequent analyses.

### Identification of differentially expressed (DE) genes and transcriptional responses across populations

We used the Bioconductor package LIMMA [31] in R to perform multiple linear regression at each gene with 1,25(OH)_2_ D3 treatment as the variable of interest. Covariates including batch, population, age, gender, % lymphocyte inhibition and cell type (% of total PBMCs) were included in the regression model. False discovery rate (FDR) was estimated using the Q-value function in R. To assess for difference in transcriptome-wide response between populations, we used LIMMA to fit a linear regression model at each gene with log_2_ fold change regressed on population. FDR was estimated as described above. In order to summarize overall transcriptional response to vitamin D treatment, we used the prcomp function in R to perform principal components analysis.

### Gene set enrichment and pathway analyses

Using the publicly available online program DAVID [32] and the commercial software IPA (Ingenuity®, www.ingenuity.com), we tested for an enrichment of DE genes in specific gene sets or canonical pathways and gene interaction networks. The significance of pathway and network analysis was controlled by one-tailed Fisher’s Exact test at p-value <0.05. As background for this analysis, we used all genes expressed in PBMCs. We performed analyses for 1,25(OH)_2_ D3 treatment alone, considering up- and down-regulated DE genes separately. To investigate the gene sets and biological pathways that are shared between both 1,25(OH)_2_ D3 and GC treatment, we included only genes that were significantly DE in both treatments. For analyses of biological pathways that play different roles in each treatment, we included only DE genes that were non-overlapping between the two treatments. Significance of overlapping genes was determined using chi-square tests.

## Results

### Suppression of PHA-mediated cellular proliferation

We previously showed that dexamethasone strongly inhibited PHA-mediated cellular proliferation and that, on average, this inhibition was less marked in African Americans [14]. To determine the extent to which vitamin D inhibits proliferation and to characterize the inter-individual and inter-ethnic variation in this cellular response phenotype, we treated these same 18 samples with 1,25(OH)_2_ D3 for 48 hours and found significant inhibition of PHA-mediated proliferation at 48 hours (mean log_2_ fold change=-0.47, p=0.0003), albeit lower than inhibition with dexamethasone treatment (mean log_2_ fold change=-3.9, p=4.8 x 10^-15^) [14]. We tested whether covariates were associated with inhibition in response to vitamin D and found that inhibition was not affected by age, gender, batch, baseline levels of *VDR* and *CYP24A1* (i.e., in the control-treated aliquot), nor serum 25(OH) D levels (all p-values≥0.05). We noted that serum 25(OH) D levels differed significantly between populations (p=0.007) and therefore we included this covariate in all subsequent analyses. We also tested whether cell type composition affected inhibition and noted that only the monocyte fraction was significantly positively correlated with inhibition of lymphocyte proliferation (Figure S1, p=0.03). In contrast to our results with dexamethasone, inhibition did not differ between AA and EA (Figure S2, p=0.58) with 1,25(OH)_2_ D3 treatment.

**Figure 1:**
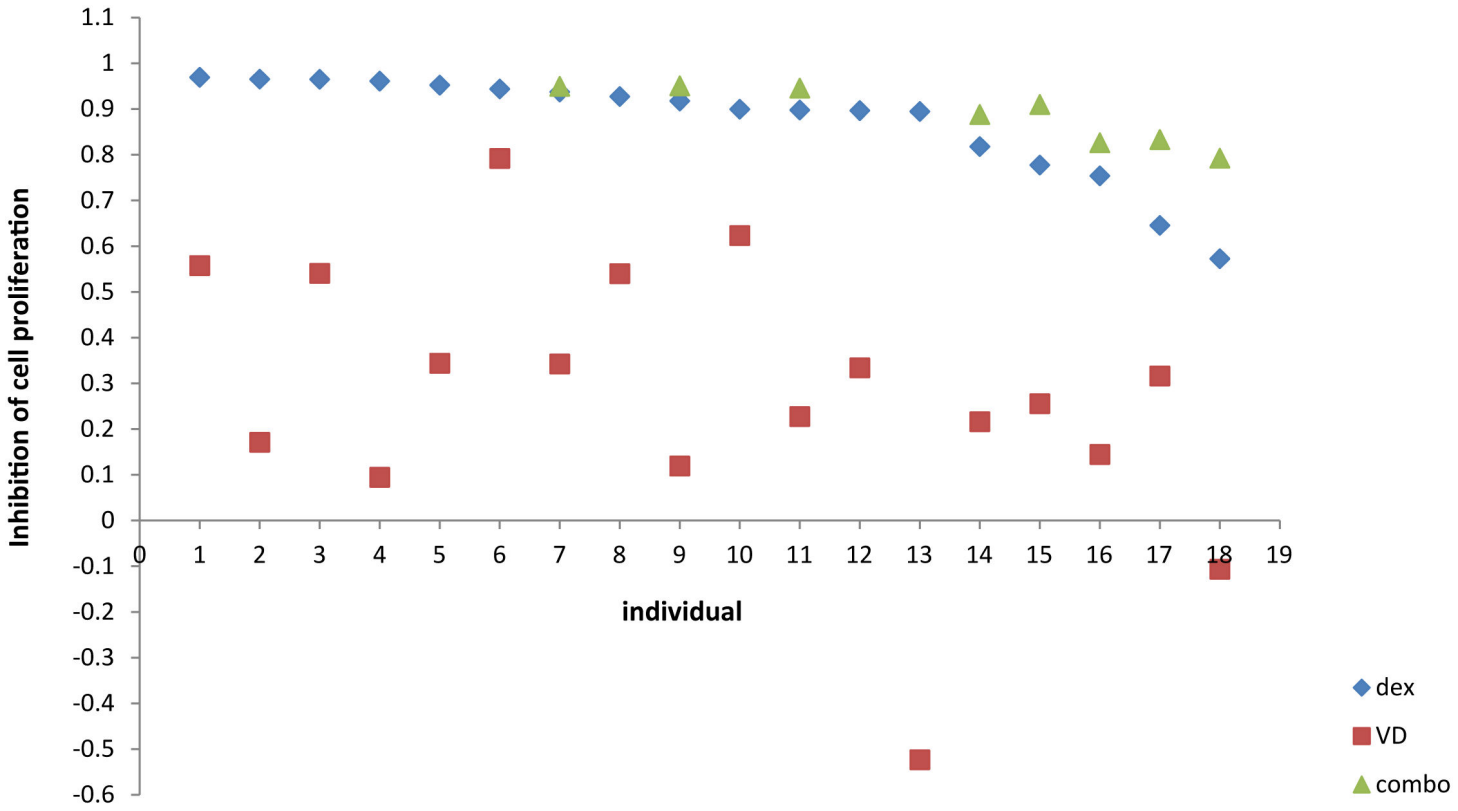
Scatterplot of inhibition of cell proliferation by individual and treatment. All individuals were treated with dexamethasone (diamond) and 1,25 vitamin D (square). Eight individuals who were least responsive to dexamethasone and for whom sufficient cells were available were treated with both dexamethasone and 1,25 vitamin D (triangle). Individuals treated with both hormones had higher % inhibition compared to either treatment alone.

Next, we wanted to learn about the effect of combined treatment in individuals with the weakest response to GC treatment alone. We selected 8 subjects with the weakest response to dexamethasone treatment (for whom sufficient cells were available) and treated their PBMCs again with dexamethasone or 1,25(OH)_2_ D3 as well as with both hormones. Here, we noted significantly greater inhibition with the combination treatment than with either steroid hormone alone (Figure 1). This difference was more pronounced for combined treatment versus 1,25(OH)_2_ D3 alone (fold change=8.22, p=1.20X10^-5^), but was also significant for combined treatment versus dexamethasone alone (fold change=1.78, p=1.81X10^-4^). While there was some variability in the degree of response across the different treatment groups, all individuals exhibited a consistent pattern of decrease in the inhibitory response going from 1,25(OH)_2_ D3 to dexamethasone and to combination treatment.

### Transcriptome-wide responses to vitamin D

**Figure 2:**
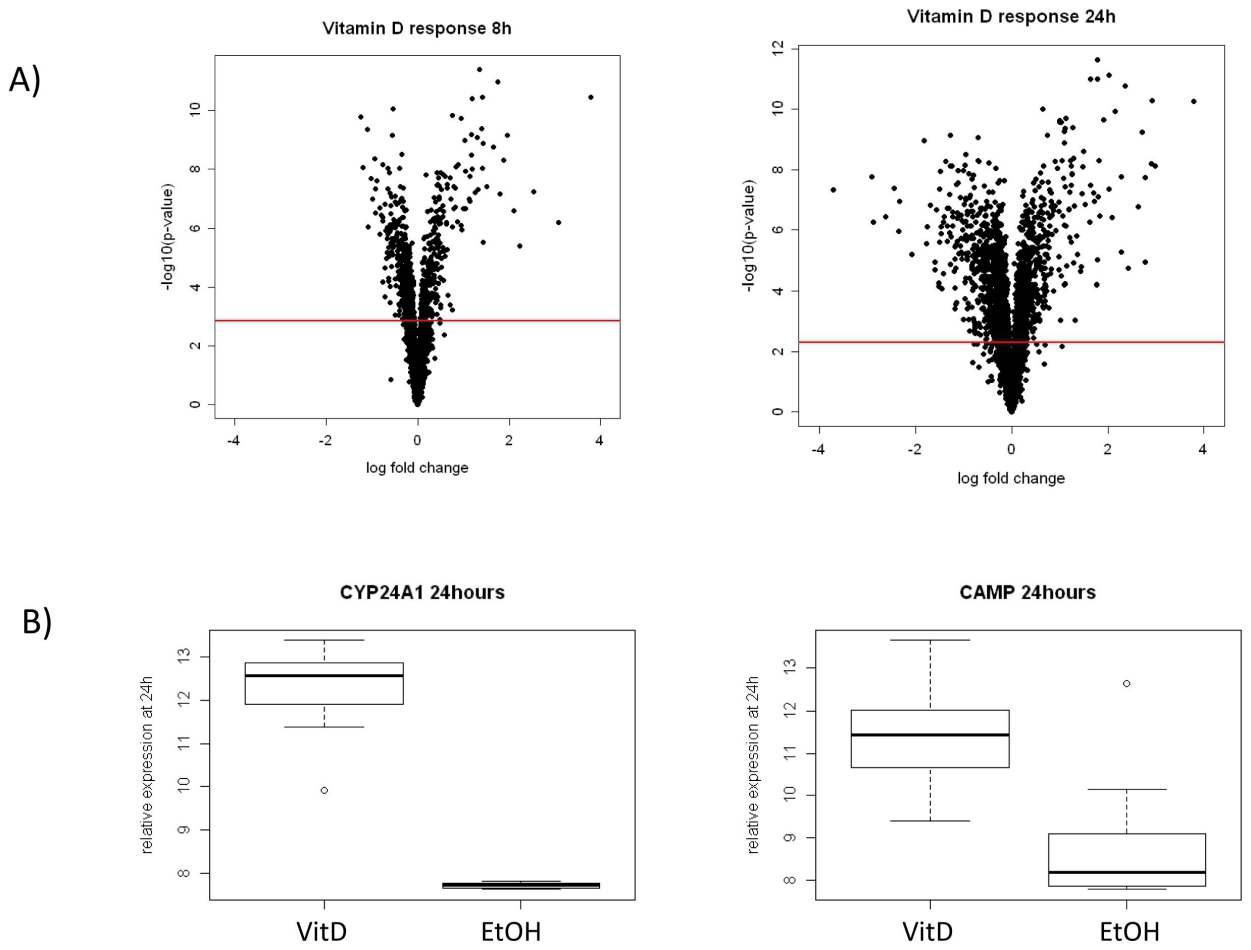
Transcriptional response to vitamin D overall and for specific vitamin D responsive genes. (**a**) Volcano plot of transcriptional response to vitamin D at 8 hours and 24 hours respectively summarizing mean log_2_ fold change (vitamin D/vehicle) and corresponding evidence of differential expression (-log_10_ p-value) for each gene shown as a single point. The red line corresponds to an FDR of 1%. (**b**) Boxplots showing examples of two established vitamin D responsive genes at 24 hours including *CYP24A1* and *CAMP*. *CYP24A1* encodes 1,25-dihydroxyvitamin D 24-hydroxylase, a member of the cytochrome P450 superfamily, that initiates degradation of 1,25 vitamin D and had the largest response to vitamin D treatment (log_2_ fold change 4.54, p-value=2.40 x 10^-9^). *CAMP*, cathelicidin anti-microbial peptide, is a known target of the vitamin D receptor and also showed a large response to treatment (log_2_ fold change 2.62, p-value=1.64 x 10^-7^).

We previously reported on transcriptional response to dexamethasone in the same assay described above and found, respectively, 2,245 and 3,373 DE genes at 8 hours and 24 hours at FDR<1% [14]. Here, we profiled the transcriptional response to 1,25(OH)_2_ D3 treatment at these same time points in the same 12 individuals (a subset of the 18 tested for lymphocyte proliferation). In samples treated with 1,25(OH)_2_ D, 3,888 and 2,271 genes were DE at 8 hours (Figure 2a, FDR<0.01) and 24 hours (Figure 2a, FDR<0.01), respectively. DE genes in response to vitamin D included some well-established VDR targets including *CYP24A1* and *CAMP* (Figure 2b)*.*

In order to determine the relationship between overall transcriptional and cellular responses, we applied principal component analysis (PCA) to the log_2_ fold change at both time points. As previously noted, the transcriptional response at 8 hours to dexamethasone treatment, as summarized by the first principal component (PC1), was correlated with inhibition of lymphocyte proliferation both within and between populations [14]. For vitamin D treatment, a similar analysis found that the PC1, which accounted for 59.9% and 75.0% of the overall variance at 8 hours and 24 hours respectively, was not associated inhibition of lymphocyte proliferation or inter-population differences in transcriptional response at 8 and 24 hours.

**Figure 3:**
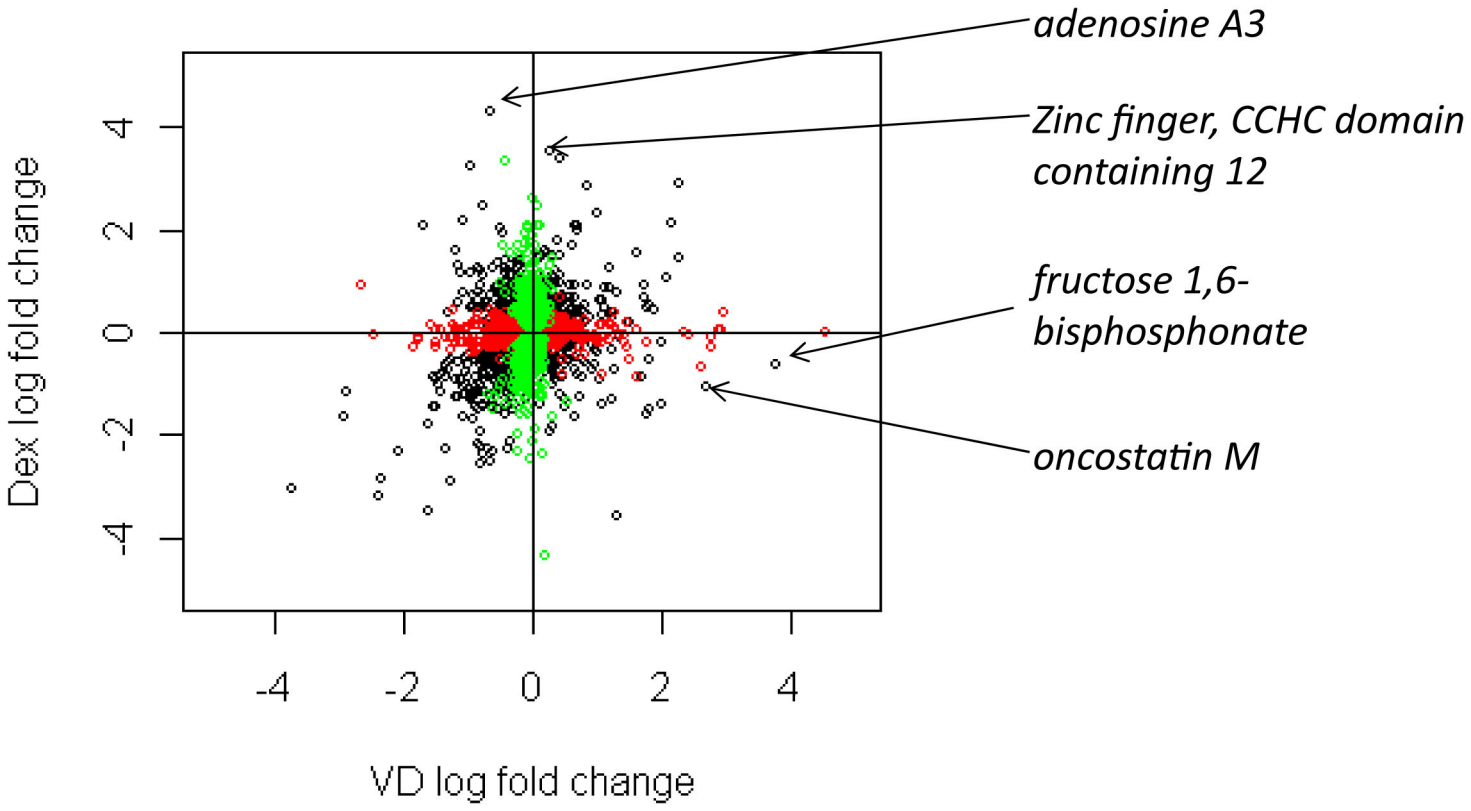
Scatterplot showing log_2_ fold change of differentially expressed genes in response to vitamin D (VD) and dexamethasone (dex) on the x- and y-axes respectively after 24 hours. Each circle represents an individual gene. Black circles indicate genes that are differentially expressed in response to *both* 1,25 vitamin D and dexamethasone treatment. Red circles are genes that are differentially expressed only in response to 1,25 vitamin D treatment; while, green circles are genes that are differentially expressed only in response to dexamethasone treatment. Names of select differentially expressed genes are indicated that show marked differences in response to 1,25 vitamin D and dexamethasone at 24 hours including fructose 1,6 *bisphosphonate* (log_2_ fold change 3.79 vs. -0.61 in vitamin D vs. dexamethasone, respectively), *oncostatin* *M* (log_2_ fold change 2.71 vs. -1.07 respectively), *adenosine* *A3* (log_2_ fold change -0.66 vs. 4.31 respectively), and *zinc* *finger, CCHC* *domain* *containing 12* (log_2_ fold change 0.27 vs. 3.51 respectively).

We then compared the transcriptional responses to dexamethasone and to vitamin D. We found a large number of genes, 2,289 and 3,296 at 8 and 24 hours respectively, that were DE in response to only one of these two treatments at the same FDR, indicating that there are significant differences in the transcriptional response to these two hormones (Figure 3). At 8 hours, there were 466 and 1,823 genes that were DE in response to only vitamin D or dexamethasone, respectively; while, at 24 hours, there were 1,097 and 2,199 genes that were DE in only vitamin D or dexamethasone, respectively. We also found many genes that overlapped between the two treatments: at 8 and 24 hours, there were, respectively, 422 and 1,174 genes that were significantly DE in response to both treatments which was significantly greater than the overlap expected by chance (p-value for overlap <0.001). Among these overlapping genes, there were a few that stood out as having different magnitudes of transcriptional response to vitamin D compared to dexamethasone and vice versa. For example, at 24 hours, fructose 1,6-*bisphophatase* (*FBP1*) and oncostatin M (OSM) showed larger log_2_ fold changes in response to vitamin D compared to dexamethasone (vitamin D vs. dexamethasone: *FBP1* 3.79 vs. -0.61, respectively and *OSM* 2.71 vs. -1.07, respectively). Whereas, *adenosine A3 receptor* (*ADORA3*) and *zinc finger, CCHC domain containing 12* (*ZCCHC12*) showed larger log_2_ fold changes in response to dexamethasone compared to vitamin D (dexamethasone vs. vitamin D: *ADORA3* 4.31 vs. -0.66, respectively and *ZCCHC12* 3.51 vs. 0.27, respectively).

### Gene set enrichment and network analysis

We previously found that a number of immune-related pathways are down-regulated in response to dexamethasone treatment consistent with the immune-suppressive effects of GCs (Table S2) [14]. To learn more about the pathways that are affected by 1,25(OH)_2_ D3 treatment as well as to the explore overlap of pathways affected by both 1,25(OH)_2_ D3 and GC treatments, we performed gene set enrichment analyses of DE genes. We did not find a significant enrichment of biological processes among vitamin D responsive genes at the 8 hour time point. However, at the 24 hour time point, we noted a significant enrichment for immune-related processes among the down-regulated genes and for cellular respiration/electron transport chain among the up-regulated genes in response to 1,25(OH)_2_ D3 treatment (Table S3).

**Figure 4:**
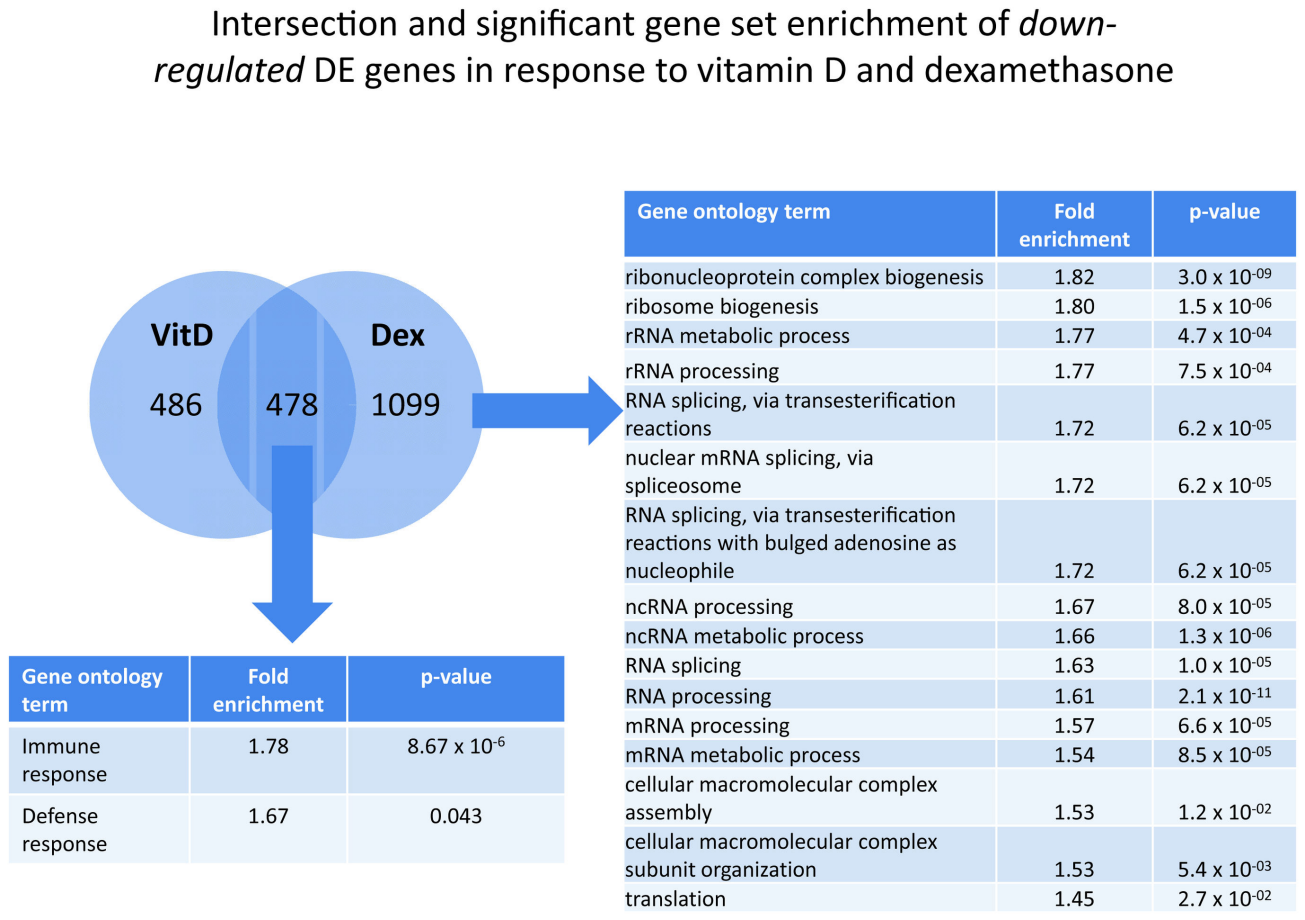
Intersection and significant gene set enrichment analysis of differentially expressed *down-regulated* genes in response to dexamethasone and vitamin D at 24 hours. Overlapping down-regulated genes were enriched for terms including “immune response” and “defense response”. Non-overlapping down-regulated genes unique to dexamethasone treatment included terms involved in RNA processing and function.

To learn about the biology underlying the increased inhibition of cell proliferation in the combined treatment relative to the single treatments, we intersected DE genes that were either up- or down-regulated in response to both single treatments. At 8 hours, there were 181 down-regulated and 77 up-regulated genes that were common to both single treatments which is significantly different than what would be expected by chance(chi-square p-values <0.001); however, these genes were not significantly enriched for any of the biological processes tested. At 24 hours, we found 478 down-regulated and 349 up-regulated genes that were common to both dexamethasone and 1,25(OH)_2_ D3 treatments (Figure 4, chi-square p-values both <0.001). In gene ontology analysis, overlap of up-regulated DE genes did not reveal a significant enrichment. On the other hand, overlapping down-regulated genes at 24 hours were significantly enriched for immune-related pathways including “immune response” (fold enrichment 1.78, p-value 6.79 x 10^-6^) and “defense response” (fold enrichment 1.67, p-value 0.04) (see Table S4a and b for details). IPA analysis of these overlapping down-regulated genes showed significant enrichment for a number of pathways (Table S5). The most significantly enriched pathway was “interferon signaling” (p-value=2.19 x 10^-9^) with a number of genes down-regulated in both IFN-gamma and IFN-alpha/beta signaling (Figure S3).

**Figure 5:**
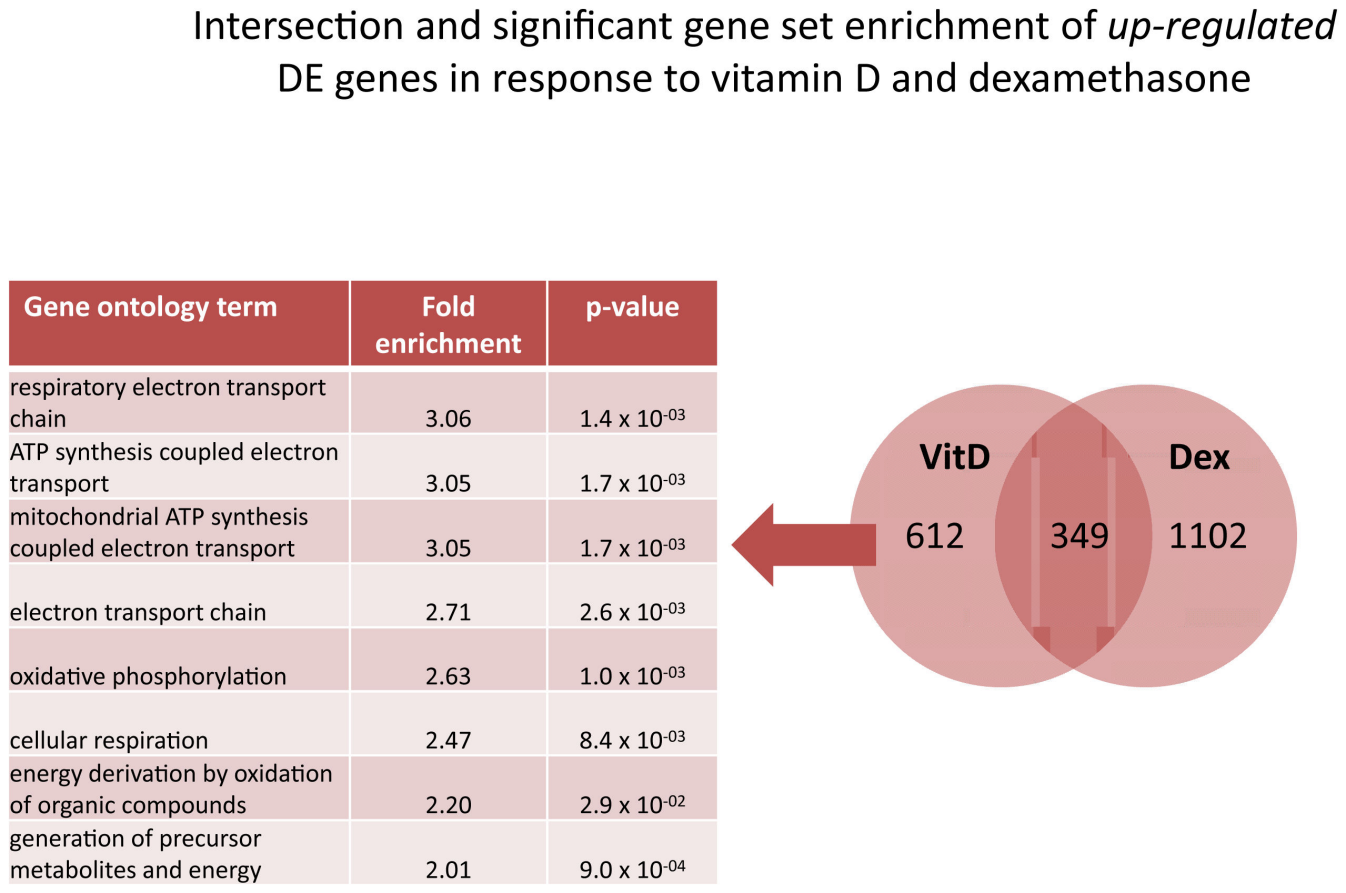
Intersection and significant gene set enrichment analysis of differentially expressed *up-regulated* genes in response to dexamethasone and vitamin D at 24 hours. Non-overlapping up-regulated genes unique to vitamin D treatment were significantly enriched for terms involved in metabolism and cellular respiration.

Next, to learn about the potential biological mechanisms of an enhanced effect of the combination treatment, we asked whether there was a significant enrichment for pathways in up- or downregulated DE genes that did not overlap between dexamethasone and 1,25(OH)_2_ D3 treatments. At the 8 hours time point, there was no enrichment for either up- or down-regulated genes for either treatment. At 24 hours of dexamethasone treatment, using gene ontology analysis, we found a significant enrichment for a number of RNA-related pathways among non-overlapping down-regulated genes (Figure 4). Network analysis of these down-regulated genes found “tRNA charging” (p=1.70 x 10^-7^) as the most significantly enriched pathway (Table S6). There was no enrichment for up-regulated DE genes in response to dexamethasone at 24 hours. For non-overlapping DE genes that were up-regulated in response to vitamin D treatment at 24 hours, we noted a significant enrichment of processes related to cellular respiration using gene ontology analysis (Figure 5). Network analysis of these up-regulated genes found “mitochondrial dysfunction” to be the most significantly enriched (p=3.02 x 10^-11^, Table S7). Among non-overlapping down-regulated genes in response to vitamin D, KEGG analysis noted “role of NFAT in regulation of the immune response” to be the most significantly enriched pathway (p=3.98 x 10^-8^, Table S8).

## Discussion

GCs and 1,25(OH)_2_ D3 have similar biological effects leading to enhanced anti-inflammatory response. However, the specific mechanisms and pathways influenced by these two hormones have not been compared. Because steroid hormones exert many of their biological actions through transcriptional mechanisms, studying transcriptome-wide expression levels in response to treatment could improve our understanding of how these hormones work in concert. In the present study, we have shown that treatment with dexamethasone plus 1,25(OH)_2_ D3 led to greater inhibition of PHA-stimulated cellular proliferation than treatment with either steroid hormone alone. Moreover, using gene set enrichment and network analyses, we found shared and non-shared pathways suggestive of potential mechanisms mediating a combined effect. We speculate that our results explain both *in vitro* and clinical observations of enhanced effects of treatment with both hormones. Moreover, understanding these shared and non-shared pathways will inform future functional and clinical studies in order to maximize combination therapy of immune-mediated diseases such as asthma, inflammatory bowel diseases and psoriasis.

Our previous work confirmed that GCs markedly inhibit proliferation of PHA-stimulated PBMCs from healthy individuals after 48 hours of treatment *in vitro* [14]. Moreover, lymphocytes from AA individuals showed weaker inhibition in response to dexamethasone than did cells from individuals of European ancestry, and these differences in cellular response were correlated with differences in transcriptional response. Treating PBMCs from these same individuals with 1,25(OH)_2_ D3 alone for 48 hours also showed an inhibition of cell proliferation, though to a lesser degree than treatment with dexamethasone. It is possible that inhibition was less pronounced due to the treatment time of 48 hours, as some previous studies have shown significantly greater inhibition of proliferation by 1,25(OH)_2_ D3 at later time points (72-96 hours) [33,34]. Interestingly, only the monocyte fraction of PBMCs was positively correlated with inhibition of lymphocyte proliferation possibly because this cell population is a primary target for vitamin D, and 1,25(OH)_2_ D3 inhibition of PHA-mediated lymphocyte proliferation is dependent on the presence of monocytes [35,36]. Moreover, In contrast to our results with GC treatment, we did not find evidence of differences in inhibition of cell proliferation in response to 1,25(OH)_2_ D3 between EA and AA.

We previously reported on gene expression in response to dexamethasone [14] and noted that, among down-regulated genes, there is significant enrichment for pathways involved in both adaptive and innate immune functions (Table S2), as has been previously described [35]. Here, we focused our attention on transcriptional response to 1,25(OH)_2_ D3 and noted a large number of DE genes at both 8 and 24 hour treatment time points. As expected, DE genes included a number of known VDR target genes such as *CYP24A1* and *CAMP* among others. Gene ontology analysis of down-regulated genes found enrichment for immune-related categories particularly those involving innate immunity (Table S3), a function of vitamin D that has recently gained increased attention [37]. In addition, we noted a significant enrichment for pathways involved in cellular respiration for up-regulated genes in response to vitamin D treatment. This is an interesting finding that supports a recent proteomics study in which 1,25(OH)_2_ D3 also induced increased activity in the electron transport chain in mitochondria of dendritic cells [28]. These authors suggest that increased cellular respiration by this mechanism makes dendritic cells more resistant to nutrient starvation. Determining whether these pathways are up-regulated in response to 1,25(OH)_2_ D3 in other cell types will require further investigation. In addition, future studies should determine whether there is functional evidence of increased cellular respiration supporting both transcriptional and translational evidence.

Consistent with similar levels of inhibition of cell proliferation across ethnic groups, we do not detect significant population differences in transcriptional responses to 1,25(OH)_2_ D3 treatment at either time point in PBMCs. This is in contrast with the patterns observed for dexamethasone, where both inhibition of cell proliferation and transcriptional response were significantly weaker in AA compared to EA. All samples are treated *in vitro* with a fixed amount of either dexamethasone or 1,25(OH)_2_ D3, which is likely to outweigh any inter-individual and inter-ethnic differences in circulating levels of the corresponding hormones. Indeed, we found that neither circulating cortisol levels nor 25(OH) D levels in the blood are significantly correlated with the *in vitro* response to dexamethasone or 1,25(OH)_2_ D3, respectively. Therefore, inter-ethnic differences, if present, are expected to be due to variation in the response to treatment rather than to variation in the hormone levels. Interestingly, AAs have lower circulating 25(OH) D serum levels compared to EAs, and the levels of the active form of vitamin D, 1,25(OH)_2_ D3, are tightly regulated and show no significant differences across populations [38–43]. Therefore, while we observed no inter-ethnic difference in the response to 1,25(OH)_2_ D3, it is possible that tissue-specific synthesis of 1,25(OH)_2_ D3 vitamin D from 25(OH) D via the CYP27B1 enzyme differs across ethnic groups. Previous studies have confirmed a role for local vitamin D metabolism, most notably in the maturation of dendritic cells and response to tuberculosis infection [44]. Future studies comparing cellular and transcriptional response using varying doses of 25(OH) D could elucidate the role of local 1,25(OH)_2_ D3 synthesis across populations.

Our gene set enrichment and network analyses of overlapping genes between GC and 1,25(OH)_2_ D3 treatment yielded enrichment of down-regulated genes involved in immune function as expected. A number of genes involved in inflammation were common to both steroid hormone treatments underscoring their primary role in both adaptive and innate immunity in peripheral blood. Because we measure response in PBMCs which is a mixture of different hematological cell types, we are not in a position to determine which cell types are responding to steroid hormones and if there are differences in cell-type specific responses between 1,25(OH)_2_ D3 and GCs. Future studies are needed to determine specific cell types that may be mediating enhanced combination treatment.

Identification of enriched pathways in *non-overlapping* genes in either 1,25(OH)_2_ D3 or dexamethasone treatment groups yielded interesting results that suggest additional ways in which these steroid hormones may act in concert. In particular, we noted a significant enrichment for pathways involved in RNA processing among down-regulated genes after dexamethasone treatment; this is an interesting finding especially given emerging evidence that GCs may exert part of their anti-inflammatory effects through post-transcriptional mechanisms [45,46]. For vitamin D-only responsive genes, we noted enrichment for processes involving cellular respiration among the up-regulated genes as was also found by a proteomics study using dendritic cells [28]. It is interesting to note that both the present study and the previous proteomics study found *FBP1* to be highly up-regulated by 1,25(OH)_2_ D3 treatment compared to dexamethasone treatment. Previous work has similarly identified this enzyme as being up-regulated by 1,25(OH)_2_ D3 in monocyte differentiation [47]. Future work is needed to determine how this vitamin D responsive enzyme may be involved in immune regulation.

In summary, our study has confirmed that treatment of peripheral blood cells with both 1,25(OH)_2_ D3 and dexamethasone increases cellular inhibition of proliferation from individuals who had the lowest response to GCs *in vitro*. We have described for the first time the transcriptional response to 1,25(OH)_2_ D3 in PBMCs and compared it to that observed in response to dexamethasone treatment. We have shown that genes that overlap between the two steroid hormone treatments are enriched for pathways involved in adaptive and innate immune function. Interestingly, non-overlapping DE genes unique to 1,25(OH)_2_ D3 treatment were enriched for cellular respiration involving the electron transport chain, while genes unique to dexamethasone treatment were enriched for RNA processing. Collectively, this work shows that 1,25(OH)_2_ D3 treatment can act through separate mechanisms from those used by GCs, likely through differences in transcriptional targets and through different effects on shared targets, providing additional immunosuppression in our *in vitro* model and, potentially, additional benefit in patients with immune-related diseases. 

## Supporting Information

Figure S1 **Among all cell types measured (monocytes, B cells, T cells, T helper and cytotoxic cells), the percent of peripheral blood mononuclear cells that were CD14+, a monocyte marker, was significantly correlated with inhibition of cell proliferation by 1,25 vitamin D treatment (ρ^2^=0.52, p-value=0.03).**
(TIF)

Figure S2 **Boxplot comparing the distribution of inhibition of cell proliferation between populations.** There was no difference in inhibition of cell proliferation in response to 1,25 vitamin D treatment between African Americans (AA) and European Americans (EA) (p-value=0.58).
(TIF)

Figure S3 **Enrichment of genes involved in interferon signaling among overlapping down-regulated genes in response to dexamethasone and vitamin D.**
(PDF)

Table S1 **Subject characteristics, sample collection times and cell composition.**
(XLSX)

Table S2 **Gene ontology analysis for DE down regulated genes in response to dexamethasone at 24 hours.**
(XLSX)

Table S3 **Gene ontology analysis for DE (A) up-regulated and (B) down-regulated genes in response to vitamin D at 24 hours.**
(XLSX)

Table S4 **(A) Genes in GO category "immune response" noted to be down-regulated in both dexamethasone and vitamin D treatment at 24 hours, (B) Genes in GO category "defense response" noted to be down-regulated in both dexamethasone and vitamin D treatment at 24 hours.**
(XLSX)

Table S5 **Network analysis of overlapping genes in response to dexamethasone and vitamin D treatment.**
(XLSX)

Table S6 **Network analysis of downregulated genes only in response to dexamethasone treatment at 24 hours.**
(XLSX)

Table S7 **Network analysis of upregulated genes only in response to vitamin D treatment at 24 hours.**
(XLSX)

Table S8 **Network analysis of downregulated genes only in response to vitamin D treatment at 24 hours.**
(XLSX)
