# Supplementary figures and images for: Comparison of Cellular and Transcriptional Responses to 1,25-Dihydroxyvitamin D3 and Glucocorticoids in Peripheral Blood Mononuclear Cells
Source: PLoS One. 2013 Oct 8;8(10):e76643. doi: 10.1371/journal.pone.0076643 (PMC3792986; doi:10.1371/journal.pone.0076643)

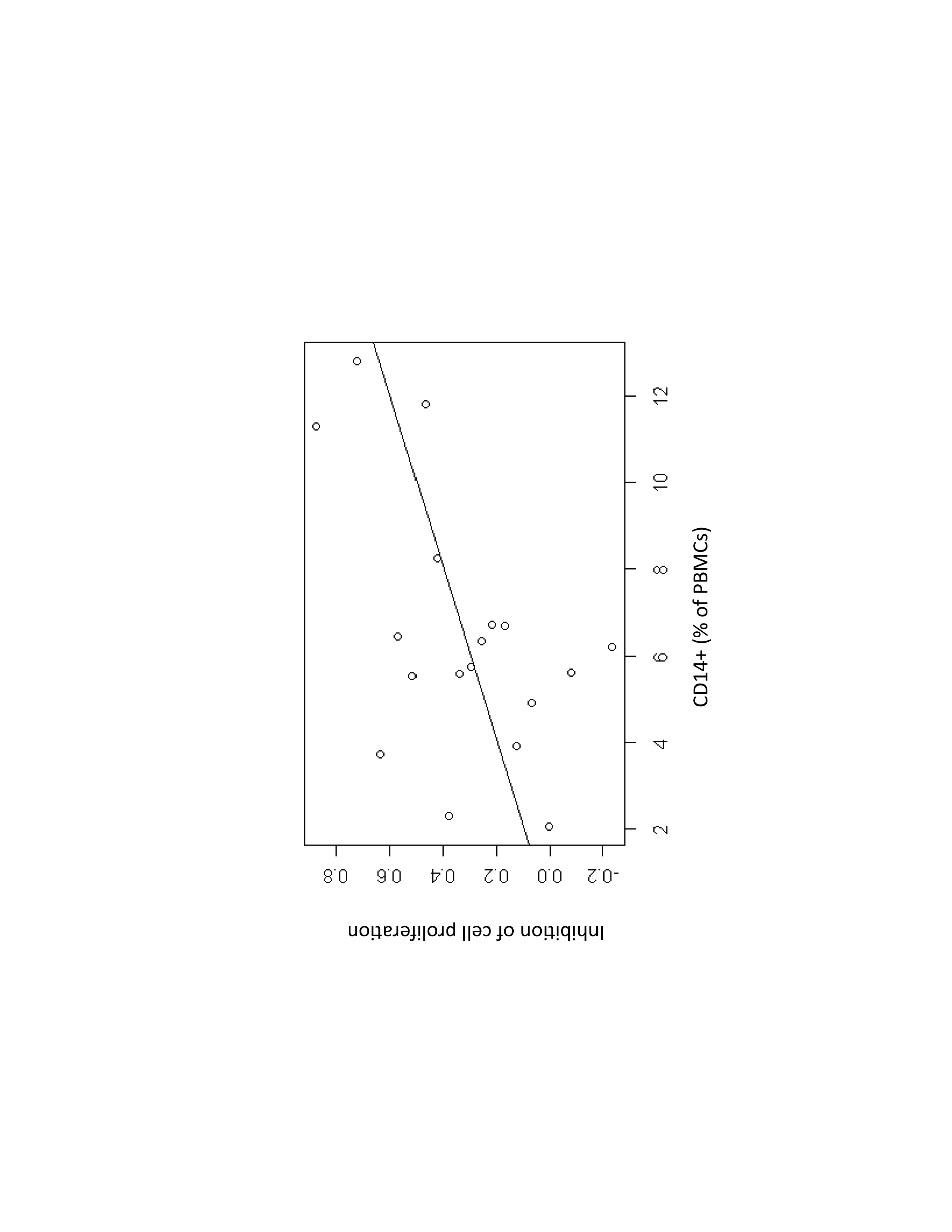

Supplement: Figure S1 — Among all cell types measured (monocytes, B cells, T cells, T helper and cytotoxic cells), the percent of peripheral blood mononuclear cells that were CD14+, a monocyte marker, was significantly correlated with inhibition of cell proliferation by 1,25 vitamin D treatment (ρ2=0.52, p-value=0.03). (TIF) [file pone.0076643.s001.tif]

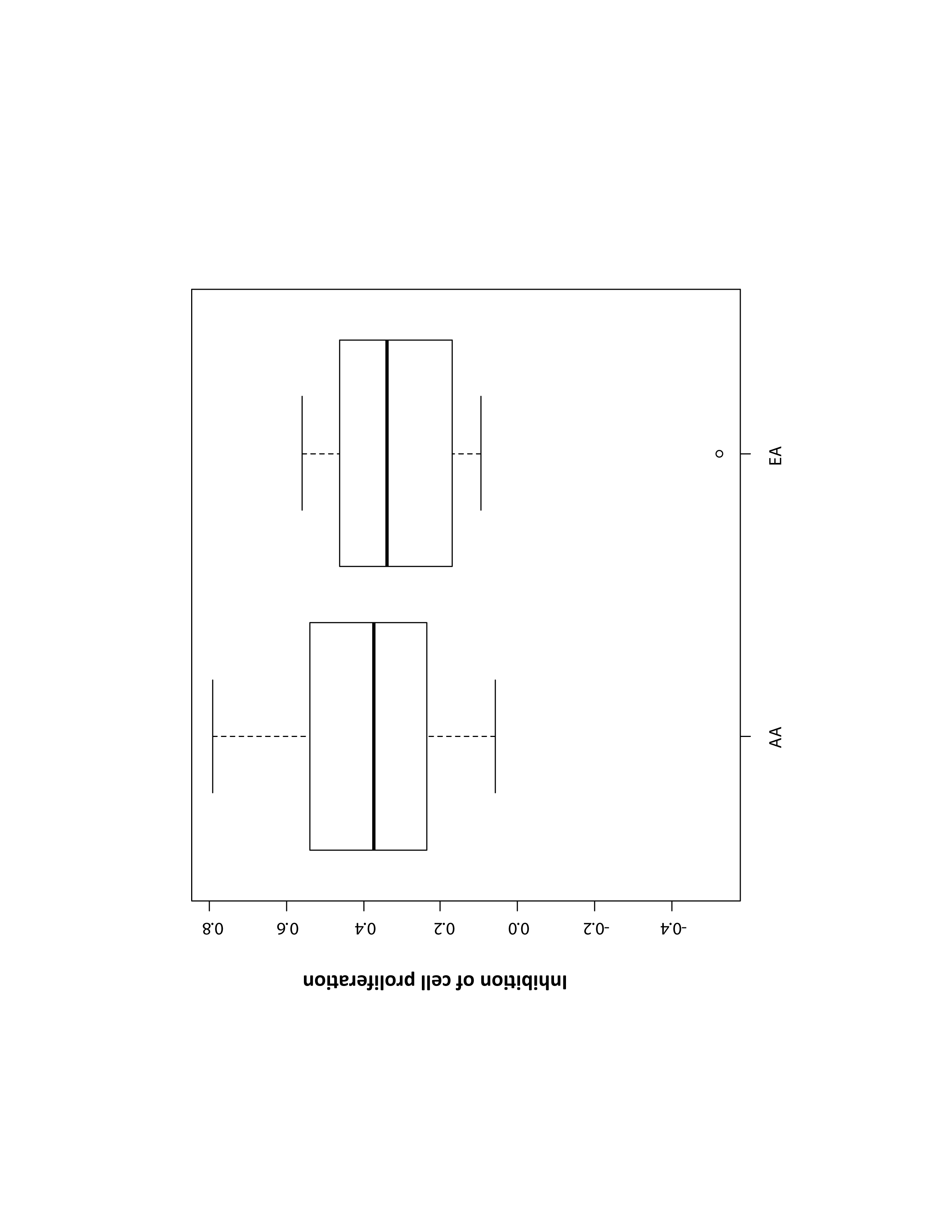

Supplement: Figure S2 — Boxplot comparing the distribution of inhibition of cell proliferation between populations. There was no difference in inhibition of cell proliferation in response to 1,25 vitamin D treatment between African Americans (AA) and European Americans (EA) (p-value=0.58). (TIF) [file pone.0076643.s002.tif]

Structural space

Topology

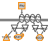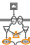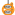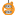

Network

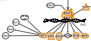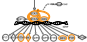

Supplement: Figure S3 — Enrichment of genes involved in interferon signaling among overlapping down-regulated genes in response to dexamethasone and vitamin D. (PDF) [file pone.0076643.s003.pdf]
